# Bacterial Community Diversity Harboured by Interacting Species

**DOI:** 10.1371/journal.pone.0155392

**Published:** 2016-06-03

**Authors:** Mikaël Bili, Anne Marie Cortesero, Christophe Mougel, Jean Pierre Gauthier, Gwennola Ermel, Jean Christophe Simon, Yannick Outreman, Sébastien Terrat, Frédérique Mahéo, Denis Poinsot

**Affiliations:** 1 Université Rennes 1, UMR1349 IGEPP, F-35000, Rennes, France; 2 Agrocampus Ouest, UMR1349 IGEPP, F-35000, Rennes, France; 3 INRA, UMR1349 IGEPP, F-35000, Rennes, France; 4 Université Européenne de Bretagne, Rennes, France; 5 UMR CNRS 6026 Interactions Cellulaires et Moléculaires, Université de Rennes, Rennes, France; 6 UMR 1347 Agroécologie, Université de Bourgogne, Dijon, France; International Atomic Energy Agency, AUSTRIA

## Abstract

All animals are infected by microbial partners that can be passengers or residents and influence many biological traits of their hosts. Even if important factors that structure the composition and abundance of microbial communities within and among host individuals have been recently described, such as diet, developmental stage or phylogeny, few studies have conducted cross-taxonomic comparisons, especially on host species related by trophic relationships. Here, we describe and compare the microbial communities associated with the cabbage root fly *Delia radicum* and its three major parasitoids: the two staphylinid beetles *Aleochara bilineata* and *A*. *bipustulata* and the hymenopteran parasitoid *Trybliographa rapae*. For each species, two populations from Western France were sampled and microbial communities were described through culture independent methods (454 pyrosequencing). Each sample harbored at least 59 to 261 different bacterial phylotypes but was strongly dominated by one or two. Microbial communities differed markedly in terms of composition and abundance, being mainly influenced by phylogenetic proximity but also geography to a minor extent. Surprisingly, despite their strong trophic interaction, parasitoids shared a very low proportion of microbial partners with their insect host. Three vertically transmitted symbionts from the genus *Wolbachia*, *Rickettsia*, and *Spiroplasma* were found in this study. Among them, Wolbachia and Spiroplasma were found in both the cabbage fly and at least one of its parasitoids, which could result from horizontal transfers through trophic interactions. Phylogenetic analysis showed that this hypothesis may explain some but not all cases. More work is needed to understand the dynamics of symbiotic associations within trophic network and the effect of these bacterial communities on the fitness of their hosts.

## Introduction

All animals harbor microorganisms, which form a community or *microbiota* [1]. If microbiota can include bacteria, fungi, virus and protozoa; bacteria are probably the most prevalent taxa both in terms of frequency and diversity [2]. This diversity involves many commensal species but also mutualists or pathogens, since some bacteria live as parasites while others improve nutrient assimilation or protect against pathogens and other natural enemies [3], [4], [5], [6]. Among animals, insects are the most diversified and ubiquitous group on Earth [7] and this is reflected by the diversity of their interactions with the bacteria they harbor. Moreover, insects provide especially good models for studying microbiota because their bacterial diversity is often simple, with typically less than 30 taxa per host [8], as compared to over 1000 taxa per host in mammals [9]. Accordingly, the interactions between insects and their microbiota have received considerable attention recently [10], [11], [12], [13], [14].

The composition of insect microbiota differs greatly between host species [15]. The most important ecological factor influencing the composition of insect bacterial communities seems to be the phylogenetic relationship between host species: closely related species often share more similar bacterial communities [16]. Within an insect species, bacterial communities can be modified by diet, developmental stage, physiological condition, geography and population structure [17], [18], [19], [20]. The varied effects of bacteria on nutrition, immunity [21], development [22], [23], reproduction [24], protection [25]) and speciation [26] indicate that bacterial symbiosis is a major component of insect fitness and evolution. In addition to the enrichment of fundamental ecological knowledge, studies of insect–bacteria interactions have produced advances in applications as diverse as biomedicine and biotechnology [27], but also aided in the identification of new targets for the control of insect pests or pathogen vectors [28].

It is estimated that 99% of bacteria cannot yet be cultivated in the laboratory [29], which represents a serious drawback to studying symbiotic bacteria diversity. However, advances in molecular biology have considerably improved culture-independent techniques to study microorganisms, thanks to PCR amplification of bacterial genes directly from environmental samples, followed by direct sequencing of PCR products [30]. Where earlier sequencing techniques provided libraries containing a few dozens of sequences, it is now possible to obtain thousands or millions of sequence reads [31]. Moreover, these sequences can be obtained using ‘universal’ bacterial 16S rDNA gene primers [32]) amplifying a great diversity of bacterium phyla, thus assessing the whole bacterial diversity of insect microbiota (at the cost of taxonomic resolution, because the 16S rDNA gene shows little variability within a given species).

Studies of microbial communities have revealed that several bacterial species are obligate symbionts of insects and strictly transmitted vertically, from mother to offspring (either within eggs or through smearing of the eggs) or horizontally, via social interactions such as trophallaxis [33], [34]. These obligate symbionts are specifically associated with several insect groups such as aphids [35], bugs [36] or social insects like termites [37]. They are located in specialized host cells called bacteriocytes, in gut crypts [38] or form large colonies in the lumen of the gut. They often provide nutrients lacking in the host diet and are essential for its survival and reproduction [3]. Some heritable symbiotic bacteria are called facultative symbionts, because they are not essential for host survival [39]. Facultative symbionts are mainly maternally inherited but sharp inconsistencies between symbiont and host phylogenies betray occasional horizontal transfers between species [24]. Facultative symbionts can induce various effects on host phenotype that increase their own vertical transmission rate to host offspring. They can raise the proportion of infected females in host populations by sexual manipulation [40] or can improve host fitness through behavioural or physiological modifications [5], [41], [42]. The best-known and studied secondary symbiont is *Wolbachia pipientis*, an *Alphaproteobacteria* widespread among different classes of arthropods and filarial nematodes [43]. Depending on strains, *Wolbachia* can benefit to some hosts but induce a range of reproductive manipulations in others, such as feminization of males, male-killing, thelytokous parthenogenesis or reproductive incompatibility between uninfected females and infected males, all phenotypes which favour maternal transmission of the infection [44]. In many insects, the majority of bacteria detected are environmentally-inherited commensals or pathogens which colonize the digestive tracts after they are consumed by insects. Various bacterial phyla are commonly present in insect guts, including *Gammaproteobacteria*, *Alphaproteobacteria*, *Betaproteobacteria*, *Bacteroidetes*, *Firmicutes* and others [45].

Most microbiota studies focus on a single host species [20], [11] or on a few phylogenetically close species [13], [15], [46], [47], [48]. They usually concentrate on host-specific bacteria [49] or on specific ecological processes [13], [14] or review several host species, which do not present any ecological relationship [16]. The objective of the present paper is to investigate the complexity of the microbiota of four species related by trophic interactions, *i*.*e*. an insect and its three main natural enemies. The cabbage root fly *Delia radicum* (*Diptera*: *Anthomyiidae*) develops in the roots of brassicaceous crops. It is mainly attacked by three solitary parasitoids: two coleopteran staphylinids (*Aleochara bilineata* and *A*. *bipustulata*) and the parasitoid wasp *Trybliographa rapae* [50]. Both *Aleochara* species develop as parasitoids of *D*. *radicum* pupae but can also prey on *D*. *radicum* eggs and larvae as adults [51], [52]. While *A*. *bilineata* is a specialist restricted to parasitizing *Delia sp*., *A*. *bipustulata* also develops using other saprophagous, necrophagous and coprophagous flies [53]. The parasitoid *Trybliographa rapae* is a strict specialist parasitizing only *Delia sp*. larvae, while adults feed on nectar like many hymenopteran parasitoids [54].

Since the bacterial communities present in the food and the environment are supposed to influence insect microbiota we expected that the bacterial communities of parasitoids exploiting the same host might be especially alike as a result of sharing the same kind of food and microenvironment during development. On the other hand, phylogeny, the level of host specificity or geography could also influence which bacterial partners are found.

Most bacteria are found in the gut lumen, but intracellular vertically transmitted facultative bacteria such as the genus *Wolbachia*, *Rickettsia* and *Spiroplasma* are a very different case. For them, host/parasitoid trophic interactions represent a gate to colonize more species during exceptional horizontal transfers. For example, laboratory experiments have shown that parasitoids are a possible transmission route for facultative endosymbionts in aphids [55] and whiteflies [56]. In the gregarious egg parasitoid of the genus *Trichogramma*, horizontal transfer of *Wolbachia* has also been observed between uninfected and infected individuals sharing the same host egg [57]. In addition, recent studies in terrestrial isopods showed that *Wolbachia* could, at low densities, cross the intestine wall and colonize a novel host [58].

Using both wild and laboratory-reared individuals, we used 454 sequencing of 16S ribosomal DNA to describe the bacterial microbiota associated with pooled DNA samples of 20–24 individual per population, with two populations per species of host (*Delia radicum*) or its parasitoids (*A*. *bilineata*, *A*. *bipustulata*, *T*. *rapae*). This constitutes to our knowledge the first description of microbiota within a trophic network. We compared bacterial communities between and within species, between each geographical location. The relative proximity of bacterial communities of the four species sampled was hard to predict. On the one hand, we assumed that parasitoids might share more bacteria with their hosts than with other parasitoids because of the intimate trophic interaction during development. On the other hand, the two *Aleochara* species are closely related [59] and share a common host species, so they might have had similar bacterial associates. Bacterial phylotypes acquired environmentally in locations where populations were initially sampled could have been conserved during rearing in the laboratory and be shared by the different species from a given location. Comparing samples could therefore allow determining whether trophic interaction, diet, geographical location or phylogenetic relation influence bacterial communities of the four species studied. Finally, 16S rDNA sequences may also outline putative horizontal transfers of intracellular symbionts such as *Wolbachia* between a host and its parasitoids.

## Materials and Methods

### Sampling, rearing and DNA extraction of insects

*Delia radicum* pupae were sampled in broccoli fields in Brittany (Western France) between April and June 2009 from three distinct areas spanning approximately 200km, which are representative of brassicaceous production: ‘Finistère’ (westernmost tip of Brittany), ‘Côtes d’Armor’ (central Brittany) and ‘Ille et Vilaine’ (easternmost Brittany) (see GPS coordinates in Table 1). For convenience, these populations will therefore be referred to as ‘western’, ‘central’ and ‘eastern’. Pupae were stored individually in punctured Eppendorf tubes in controlled conditions (20±1°C, 60±10% RH and a 16-L: 8-D photoperiod) until emergence of adults. Individuals of the four species (*D*. *radicum* and its three parasitoids *A*. *bilineata*, *A*. *bipustulata* and *T*. *rapae*) emerging from the pupae were then collected and stored in 95% ethanol (*A*. *bilineata*) or reared in the laboratory between 6 and 18 months in the same controlled conditions as above and then stored in 95% ethanol (other species). In order to study most of the extensive microbiota that individuals of each species could harbor, we took individuals at the adult stage in laboratory populations. Sampling site and rearing period for each species are summarized in Table 1. No specific permissions were required for these collection activities and samplings did not involve endangered or protected species.

**Table 1:** Details about samples: *Species*, *sites sampled*, *corresponding area*, *number of generations reared in the laboratory and* number of individuals used in 454 sequencing.

Species	Sampling site (geographical coordinates)	Lab. generations	Nb. ind.
*Delia radicum*	Western (one field: 48°40’N, 3°59W)	6	20
*Delia radicum*	Eastern (one field: 48°06’N, 1°47’W)	18	20
*Aleochara bilineata*	Western (several fields, 48°40’N, 3°55W)	Wild individuals	20
*Aleochara bilineata*	Central (several fields, 48°60’N, 1°88W)	Wild individuals	20
*Aleochara bipustulata*	Western (one field: 48°40’N,3°50’W)	12	20
*Aleochara bipustulata*	Eastern (one field: 48°06’N, 1°47’W)	12	20
*Trybliographa rapae*	Western (several fields, 48°40’N, 3°55W)	3	24
*Trybliographa rapae*	Eastern (one field: 48°06’N, 1°47’W)	9	24

### Rearing

*D*. *radicum* populations were maintained on Swede roots (*Brassica napus*) following a method derived from Keymeulen et al. [60]. All parasitoids were raised using the western population of *D*. *radicum* as a host. *A*. *bipustulata* adults were kept in plastic boxes (16×9.5×8 cm) filled up with moistened vermiculite and containing *D*. *radicum* pupae as a host and minced beef *ad libitum* as a food source. Once a week, parasitized pupae were collected from the rearing box and stored in another one with moistened vermiculite until parasitoid emergence. *Trybliographa rapae* populations were maintained using the rearing conditions described in Neveu *et al*. [61] and were offered *D*. *radicum* larvae as a host.

### DNA extraction

Individuals from *A*. *bilineata* had been stored in 95% alcohol immediately upon emergence from wild-collected *D*. *radicum* pupae. In other species, 20–24 individuals were directly taken at random from laboratory populations and placed together in 95% ethanol in an Eppendorf tube. Individuals were surface-sterilized with bleach diluted in ultrapure water at 3% for 1 minute and rinsed 3 times for 1 minute with sterile ultrapure water. Then, total DNA was extracted using the DNeasyTM Tissue Kit (Qiagen SAS, Courtaboeuf, France) following the standard protocol provided. Due to differences in insect sizes, extraction took place for each population in two distinct Eppendorf tubes containing each 10–12 individuals for the three small parasitoid species and in ten Eppendorf tubes containing each only two individuals for their larger host, *D*. *radicum*. DNA extractions for each sample were quantified and normalized using a Nanodrop spectrophotometer and DNA extractions were combined to create a single DNA pool per population.

### Bacterial 16S rDNA gene PCR amplification

For each DNA pool (one pool = one population of one species), two independent PCR took place (and were later sequenced independently). A ~420bp fragment of the v4-v5 region of the bacterial 16S rDNA gene was amplified using the universal bacterial primers Fwd: GTGCCAGCMGCCGCGGTAATAC—3’ and rev: CCGTCAATTCCTTTGAGTTT—5’ from Yu & Morrisson [62]. Primers were modified by the addition of a GS FLX Titanium Key-Primer A and B (A: CGTATCGCCTCCCTCGCGCCATCAG and B: CTATGCGCCTTGCCAGCCCGCTCAG) and a multiplex identifier (MID) sequence specific to each species. The MID sequences were as follow for each species: *D*. *radicum* (GAGTA/TACTG), *T*. *rapae* (GACGT/GTCTC), *A*. *bipustulata* (TGTAC/TGTAC) and *A*. *bilineata* (TCTCG/TGACT). Thus, the two independent PCR products per pool shared the same MID. The PCR reaction mixture (50 μL) consisted of 0.356 μL of DNA polymerase (3.5 U/μL), 2 μL of each primers (10 μM), 2.5 μL of dNTPs (10 mM each), 5 μL of 10 X PCR buffer (20 mM Tris-HCl, pH 8.4, 50 mM KCl and 1.75 mM MgCl_2_), and 2 μL of extracted DNA completed by 36.144 μL of ultrapure water. The PCR parameters were 94°C for 2 min; 15 cycles of 94°C for 30 s, 64°C for 30s and 72°C for 1min; 20 cycles of 94°C for 30 s, 58°C for 30 s and 72°C for 1 min; and a final step at 72°C for 6 min. Each PCR product was then cleaned using the QIAquick PCR purification kit (Qiagen) following the manufacturer’s instructions.

### 454 pyrosequencing

PCR products were sequenced, using 454 pyrosequencing (Environmental genomics platform of ‘Biogenouest’, Rennes, France. For each sample, PCR products were used in two independent emulsions and sequenced in both directions independently in one run using the protocol GS-FLX-Titanium Amplicon sequencing provided by Roche. The sequences generated by the present study have been deposited in the European Nucleotide Archive (ENA) and are available at: http://www.ebi.ac.uk/ena/data/view/PRJEB12751.

### Sample assignments and analysis of 454 sequencing data

All 16S rDNA gene pyrosequencing reads were analyzed using the GnS-PIPE developed by the GenoSol platform (INRA, Dijon, France) and optimized for amplicons analysis [63], [64]. First, all the raw reads were sorted according to the multiplex identifier sequences. The raw reads were then filtered and deleted if they harbored: (a) a length lower than 400 bp, (b) one or more ambiguities (Ns), (c) an error in the proximal primer sequence (the distal primer sequence must be perfect too, but can be potentially incomplete), (d) an average quality score below 20. A PERL program was then applied for rigorous dereplication (i.e. clustering of strictly identical sequences). The dereplicated reads were then aligned using Infernal alignment [65], and clustered into Operational Taxonomic Units (OTUs) at 97% of similarity using a PERL program that groups rare reads to abundant ones, and does not count differences in homopolymer lengths. A filtering step was then carried out to check all single-singletons (reads detected only once and not clustered, which might be artifacts, such as PCR chimeras) based on the quality of their taxonomic assignments. Finally, in order to compare the datasets efficiently and avoid biased community comparisons, the reads were homogenized by random selection of 3,000 reads for each sample. The retained high-quality reads were used for: (i) taxonomy-independent analyses, determining several diversity and richness indexes (number of clusters, Chao1 estimator, Shannon and Evenness) using the defined OTU composition, and (ii) taxonomy-based analysis of each OTU using similarity approaches against dedicated reference databases from SILVA [66]. The bacterial communities from all samples were also compared and clustered by using unweighted UniFrac distances [67], based on the 16S phylogenetic trees computed with FastTree [68]. More precisely, UniFrac distances are a measure of the phylogenetic distance between sets of taxa (in a phylogenetic tree) and are useful to compare the similarity of microbial communities. Specifically, “UniFrac measures the phylogenetic distance between sets of taxa in a phylogenetic tree as the fraction of the branch length of the tree that leads to descendants from either one environment or the other, but not both” [67].

### Phylogenetic analyses of vertically transmitted bacteria

Representative sequences of OTU corresponding to bacteria known to be vertically transmitted and shared by at least two species of our sample were then aligned with mafft 6.822 using default parameters [69] and their phylogenetic positions compared with reference GenBank 16S rDNA genes from other arthropod hosts (S1 Table).

Maximum Likelihood (ML) trees were built for Rickettsia spp., Spiroplasma spp. and Wolbachia spp. First, we used the Akaike information criteria corrected for small sample sizes [70] to select the best-fitting substitution models with jmodeltest [71]. We then built ML trees for each genus using RAXML 7.2.7 [72]. Node support was assessed by bootstrapping (1000 pseudoreplicates). Ehrlichia chaffeensis (Rickettsiales: Anaplasmataceae) was added and used as outgroup for Rickettsia spp. and Wolbachia spp. ML trees; Mycoplasma hominis (Mycoplasmatales: Mycoplasmataceae) was added and used as outgroup for Spiroplasma spp. ML tree.

### Wolbachia typing using primers W-spec and fbpA

The Wolbachia variants revealed by 454 sequencing in *Delia radicum*, *Aleochara bilineata* and *A*. *bipustulata* were more precisely characterized as follows. For each DNA pool (two pools per species i.e. one per population), two independent PCR took place using the same PCR reaction mixtures and PCR parameters. In the first one, a 415bp fragment of the bacterial 16S rDNA gene was amplified using primers W-Specf—CATACCTATTCGAAGGGATAG—3’ and W-Specr: ACGTTCGAGTGAAACCAATTC—5’ [73]. In the second one, a 478bp fragment of the fbpA gene was amplified using primers fbpA_F1: GCTGCTCCRCTTGGYWTGAT—3’ and fbpA_R1: CCRCCAGARAAAAYYACTATTC—5’ [74]. The PCR reaction mixture was identical to that which was described in the part "Bacterial 16S rDNA gene PCR amplification" a final reaction volume of 25μL with the appropriate primers. The PCR parameters were 94°C for 2 min; 35 cycles of 94°C for 30 s, 55°C for 45s and 72°C for 1min; and a final step at 72°C for 10 min. The PCR products were sequenced using the Sanger method, using an ABI 3730xl capillary sequencer. Sequences were aligned and analyzed using Geneious 7.0.6. With 16s and fbpA sequences combined from Wolbachia, we then built a ML tree using RAXML 7.2.7 [72]. Node support was assessed by bootstrapping (1000 pseudoreplicates). The data were partitioned into ribosomal regions (two 16S rDNA fragments) and fbpA.

## Results

We were able to successfully amplify bacterial 16S rDNA gene sequences from all samples collected in the study. A half-run of the final pooled PCR amplicons on a 454 GS FLX Titanium Sequencer yielded a total of approximately 250,000 sequences. Post-quality checking (length, chimera, barcode, primer sequence removal and base-quality checks) resulted in 61,700 sequences of a minimum length of 400bp. Homogenization of the samples sets led to keep 48,000 sequences (3000 for each of sample).

**Fig 1:**
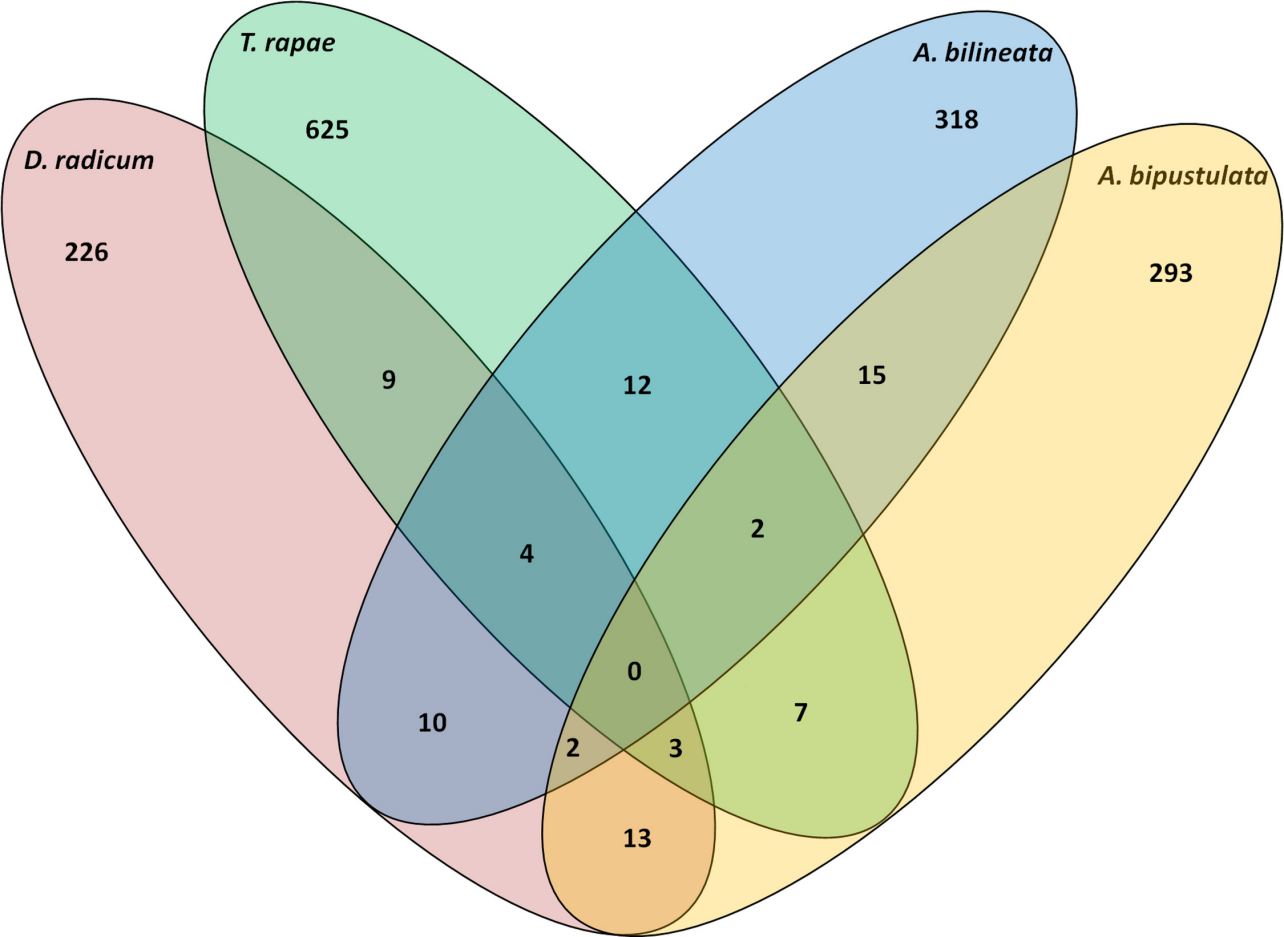
Distribution of OTUs (97% similarity) among the four host species.

Bacterial richness varied between 59 OTUs (in 9 genera) for western *D*. *radicum* (i.e. *D*. *radicum* from the ‘western’ population) and 261 OTUs (in 65 genera) for western *T*. *rapae* (Table 2). Chao1 estimator values ranged between 182 OTUs for *D*. *radicum* and 539 OTUs for *T*. *rapae*, so that the coverage of the estimated microbial diversity ranged from 30% for western *D*. *radicum* to 63% for eastern *D*. *radicum* which is coherent with other studies on insect microbial communities. Shannon indexes highlighted large differences of bacterial diversity between samples and were comprised between 0.75 for *D*. *radicum* and 3.25 for *T*. *rapae*. Evenness was relatively low and varied between 0.18 for *D*. *radicum* and 0.58 for *T*. *rapae*. Minor differences were found between diversity indices computed from the two PCR replicates of the same biological samples. No OTU was shared by all species and only a small fraction of OTUs were shared by 2–3 species (Fig 1). Out of 1506 OTUs, 1350 were represented by less than 10 sequences.

**Table 2:** Bacterial diversity indexes: Richness and diversity indexes associated with each sample and replicate. ‘Western’, ‘central’ and ‘eastern’ populations refer respectively to the Finistère, Côtes d’Armor and Ille & Vilaine regions of Brittany. Coverage = estimated percentage of (Chao 1) richness identified.

Species	Population and (PCR replicate)	Richness (nb OTU)	Chao1	Coverage (%)	Shannon	Evenness
	western (1)	68	229	30	0.76	0.18
*D*. *radicum*	western (2)	59	182	32	0.75	0.18
	eastern (1)	95	151	63	1.74	0.38
	eastern (2)	101	198	51	1.76	0.38
	western (1)	83	248	33	1.58	0.36
*A*. *bilineata*	western (2)	90	282	32	1.59	0.35
	central (1)	129	307	42	1.79	0.37
	central (2)	119	263	45	1.76	0.37
	western (1)	102	309	33	1.11	0.24
*A*. *bipustulata*	western (2)	96	218	44	1.11	0.24
	eastern (1)	109	239	46	1.22	0.26
	eastern (2)	110	216	51	1.20	0.26
	western (1)	261	539	48	3.25	0.58
*T*. *rapae*	western (2)	258	493	52	3.15	0.57
	eastern (1)	155	303	51	2.04	0.40
	eastern (2)	161	324	50	2.14	0.42

Three major phyla represented 98% of analysed sequences: *Proteobacteria* (61%), *Tenericutes* (25%) and *Firmicutes* (12%). *Bacteroidetes* (1%), and *Actinobacteria* (0.3%) were only represented by a few sequences. Within *Proteobacteria*, *Alphaproteobacteria* (49%) were the most prevalent taxon with sequences in each samples and dominating *D*. *radicum* and *T*. *rapae* bacterial communities. *Gammaproteobacteria* (11%) were mostly represented in *A*. *bilineata* samples from the central population while *Betaproteobacteria* represented only 1% of total sequences. All *Tenericutes* sequences belonged to *Spiroplasma sp*. while *Firmicutes* were represented by several genera: *Bacillus* sp. (dominating the western *A*. *bilineata* community), *Solibacillus sp*., *Lysinibacillus sp*., *Sporosarcina sp*. and *Enterococcus sp*.

Globally, regarding the taxonomy-based analysis, 108 different bacterial genera were identified in our study. For every sample, one or two genera were strongly represented while many were represented by less than 10 sequences (Table 3, cumulating the content of both PCR replicates). The less diversified bacterial community was found in *D*. *radicum* samples: the genus *Wolbachia*, a vertically transmitted symbiont, represented 97% of the sequences retrieved in the western population and 80% in the eastern population, where *Gluconobacter* represented 18%. Of the 19 genera identified in *D*. *radicum* samples (9 in the western population and 15 in eastern samples) only 5 were shared between the two populations. In the two *A*. *bipustulata* populations, the vertically transmitted symbiont *Spiroplasma* represented 90% of sequences and *Wolbachia sp*. was also detected (10% and 5% of sequences in western and eastern populations respectively). Globally, 33 genera were identified and 11 were shared between the two populations. *Aleochara bilineata* populations were quite different. In the west, the genus *Bacillus* dominated the bacterial community (75% of the reads) while in the central population it was *Stenotrophomonas* (70%). Again, a few sequences belonging to *Wolbachia* were retrieved and a sequence belonging to *Rickettsia* was also identified in the two populations. A total of 34 genera were identified and only 7 were shared between the two populations. Although *T*. *rapae* exhibited the most remarkably diversified bacterial community in our study (261 OTU) over 90% of sequences from the eastern population belonged to *Asaia* (with the genus *Azospirillum* at 5%), while in the west the hymenopteran harboured mainly the vertically transmitted symbiont *Rickettsia* (40%), *Asaia* (30%) and *Stenotrophomonas* (20%). Globally 76 genera were identified in the two populations of *T*. *rapae* and 31 were retrieved in the two populations.

**Table 3:** Abundance of bacterial taxa from each samples: Abundance of dominant species in our dataset based on 97% similarity OTU assignments. ‘Western’, ‘central’ and ‘eastern’ populations refer respectively to Finistère, Côtes d’Armor and Ille & Vilaine regions of Brittany. Numbers indicate the number of reads associated to each genus. Known heritable symbionts are marked by an asterisk (results of both PCR replicates are pooled).

*Delia radicum*		*Aleochara bilineata*		*Aleochara bipustulata*		*Trybliographa rapae*		
western	eastern	western	central	western	eastern	western	eastern	
								***Bacteroidetes***
-	1	-	-	2	-	223	32	*Flavobacterium*
-	-	-	-	2	11	55	-	*Myroides*
-	-	-	-	12	-	105	19	*Sphingobacterium*
								***Firmicutes***
5	-	4474	219	-	2	4	-	*Bacillus*
-	74	-	1	21	-	3	-	*Enterococcus*
-	-	530	3	-	-	-	-	*Lysinibacillus*
-	-	210	-	-	-	-	-	*Solibacillus*
-	-	175	3	-	-	-	-	*Sporosarcina*
								***Alphaproteobacteria***
-	9	-	-	-	1	1754	5470	*Asaia*
-	-	-	-	-	-	31	151	*Azospirillum*
-	1097	-	-	-	-	-	9	*Gluconacetobacter*
-	-	1	1	2	8	21	4	*Ochrobactrum*
-	-	-	-	1	80	16	2	*Pseudochrobactrum*
-	-	8	117	-	-	1471	-	Rickettsia*
5984	4791	218	109	577	260	3	-	Wolbachia*
								***Betaproteobacteria***
-	1	-	-	-	5	88	3	*Achromobacter*
1	2	-	-	4	3	43	2	*Deftia*
2	2	2	-	2	35	47	9	*Ralstonia*
								***Gammaproteobacteria***
-	-	-	-	-	-	51	4	*Legionella*
-	-	7	108	-	-	-	-	*Pseudomonas*
-	-	-	4210	24	1	593	32	*Stenotrophomonas*
								***Tenericutes***
1	4	138	939	5313	5516	3	-	Spiroplasma*

**Fig 2:**
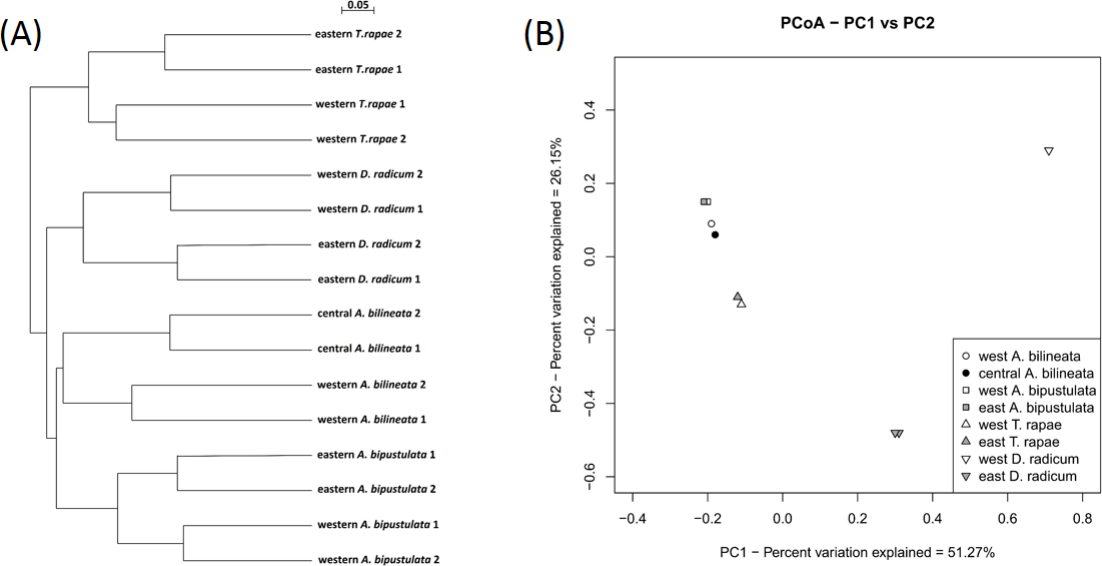
Relationship between bacterial communities via UniFrac distances. (A): Hierarchical clustering of weighted pairwise UniFrac distances between microbial communities of the different samples based on their distribution of bacterial 16S rDNA gene sequences (v4-v5 region). (B): Principal coordinates plot (PCoA) generated using weighted UniFrac distances between the bacterial communities for each sample analysed. Different shapes represent species: inverted triangle = *D*. *radicum*, upright triangle = *T*. *rapae*, square = *A*. *bipustulata*, circle = *A*. *bilineata*. Colours represent sampling zones: white = western, grey = eastern, black = central (for *A*. *bilineata* only). Two technical replicates are used, but with the exception of eastern *D*. *radicum* they are so similar as to appear superposed on the graph.

To determine if the variation in OTU diversity was significantly different between bacterial communities associated to our samples, we compared the microbial diversity using UniFrac (Fig 2). UniFrac significance with all environments together showed that our samples harbored significantly different microbiota (*p* = 0.02). Unweighted clustering with UniFrac shows the tight proximity between the two sequencing replicates of each PCR sample. Samples clearly cluster by species. The *A*. *bipustulata* cluster is sister group to the *A*. *bilineata* cluster, this *Aleochara sp*. group is sister group to the *D*. *radicum* cluster, and the *T*. *rapae* cluster occupies the most basal position.

**Fig 3:**
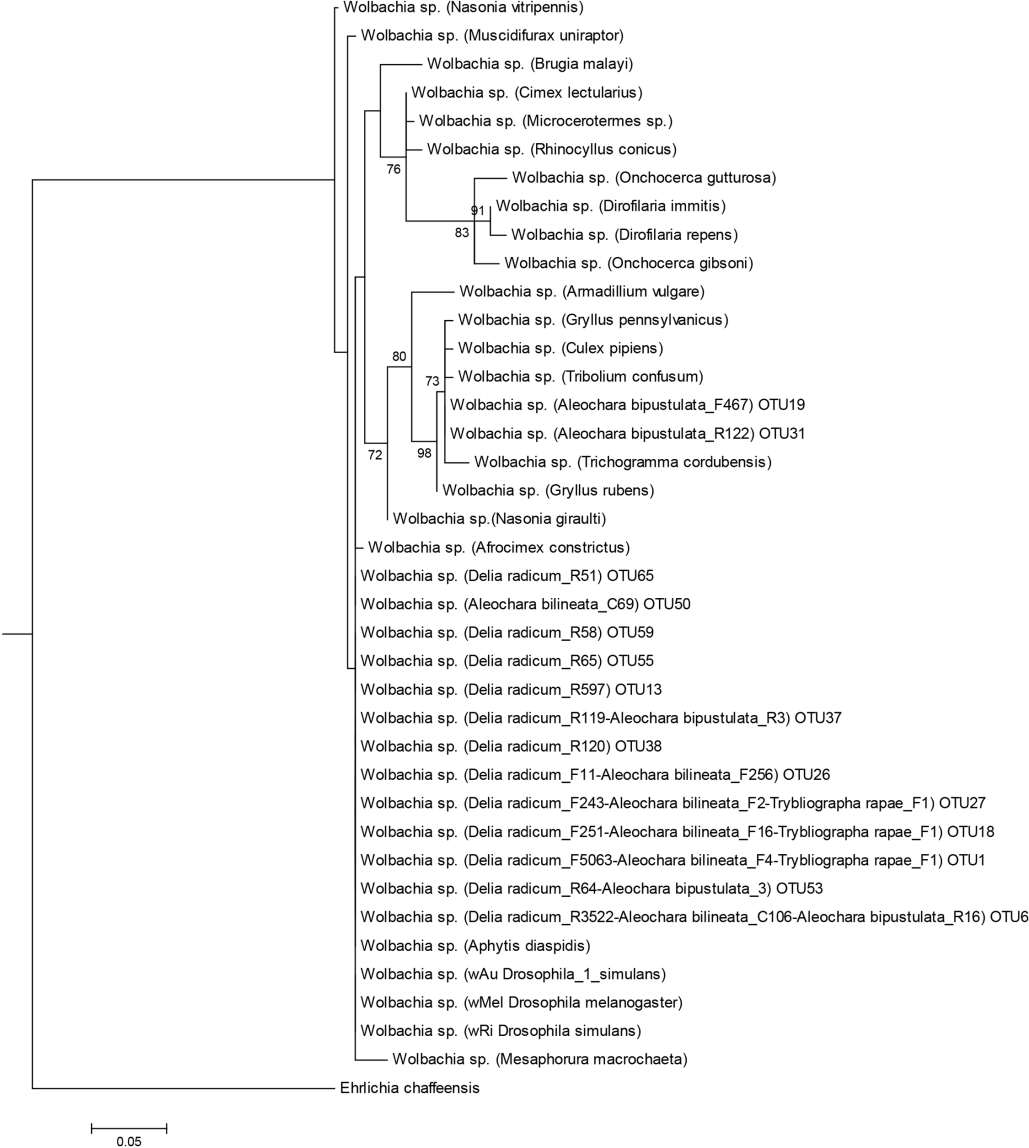
Maximum likelihood tree of Wolbachia spp. obtained from the 16S V4-V5 fragment. Information is presented in the following order: bacteria name; host name in brackets (when available); OTU number for *Delia radicum*, *Aleochara bilineata*, *Aleochara bipustulata* and *Trybliographa rapae*. Bootstrap values (≥60; black) are given for each branch. Scaling is expressed in the proportion of substituted bases per site. Capital letters A to M refer to Wolbachia ‘supergroups’.

**Fig 4:**
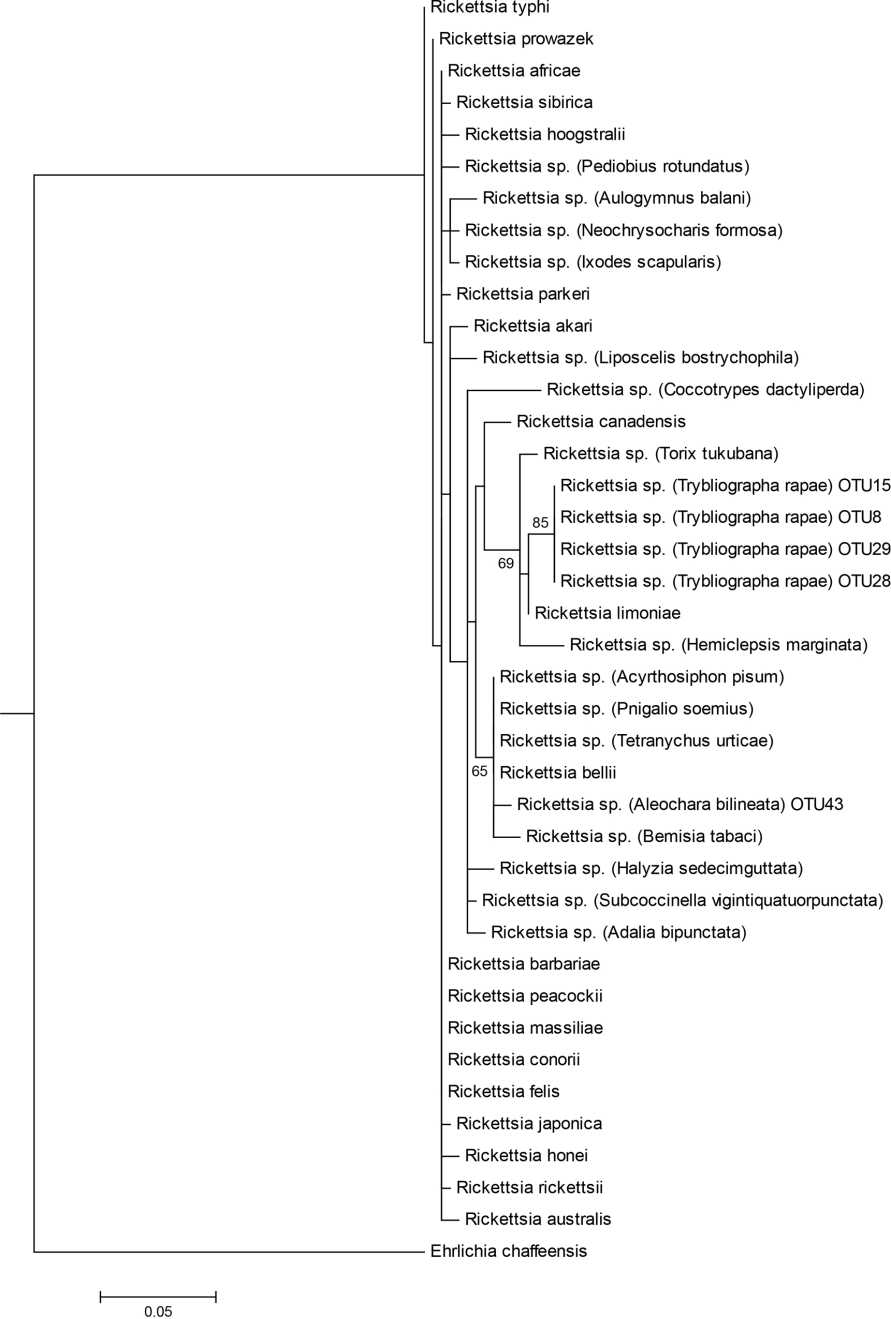
Maximum likelihood tree of Rickettsia spp. obtained from the 16S V4-V5 fragment. Information is presented in the following order: bacteria name; host name in brackets (when available); OTU number for *Aleochara bilineata* and *Trybliographa rapae*. Bootstrap values (≥60; black) are given for each branch. Scaling is expressed in the proportion of substituted bases per site.

**Fig 5:**
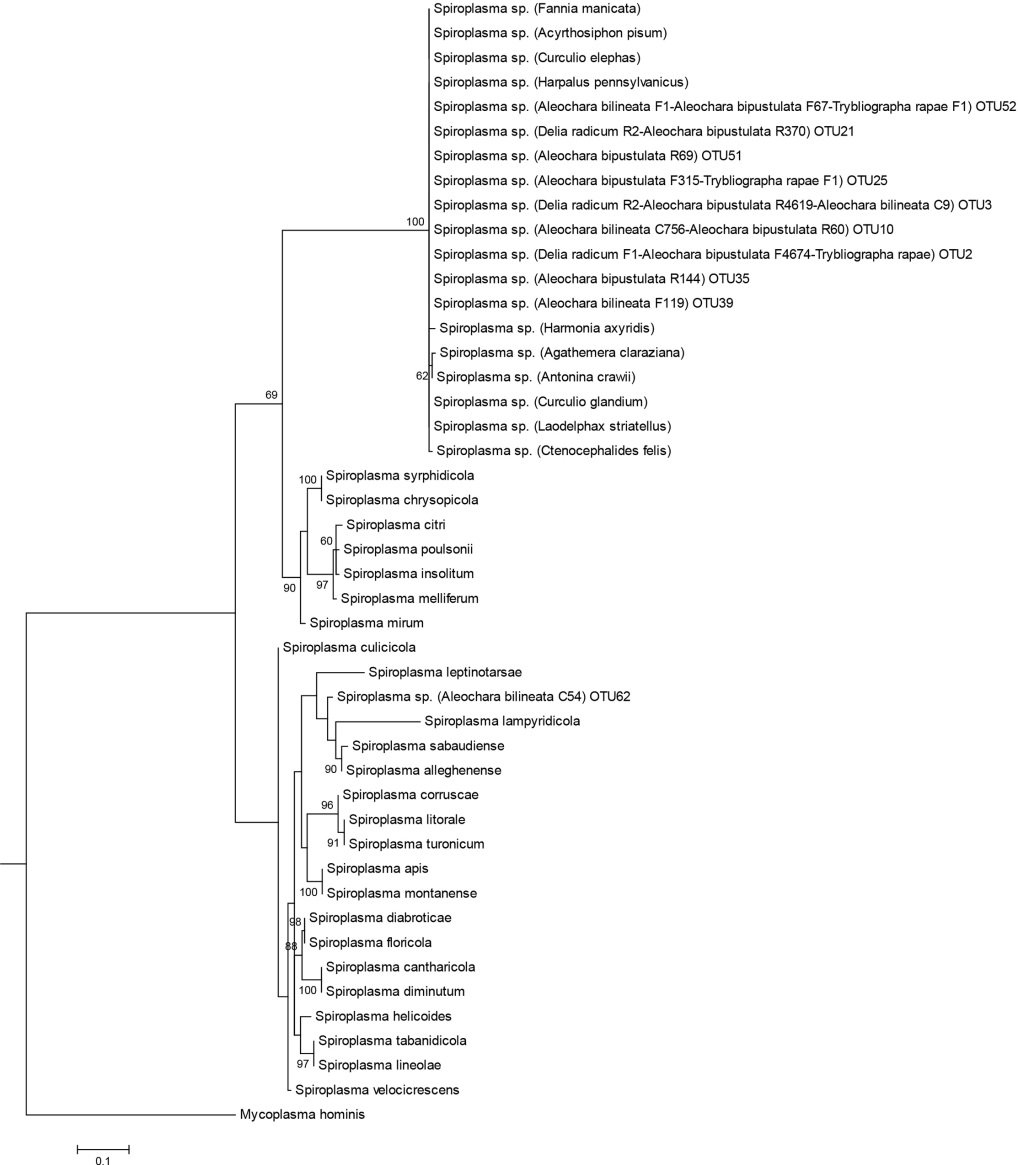
Maximum likelihood tree of Spiroplasma spp. obtained from the 16S V4-V5 fragment. Information is presented in the following order: bacteria name; host name in brackets (when available); OTU number for *Delia radicum*, *Aleochara bilineata*, *Aleochara bipustulata* and *Trybliographa rapae*. Bootstrap values (≥60; black) are given for each branch. Scaling is expressed in the proportion of substituted bases per site. Roman letters indicate serological groups.

A phylogenetic analysis of the 454 sequences was performed for the three prevalent heritable bacteria shared by two or more species: *Wolbachia* was detected in the two *Aleochara* species and *D*. *radicum*, *Rickettsia* was detected in *T*. *rapae* and *A*. *bilineata*, *Spiroplasma* was detected in the two *Aleochara* species (Figs 3, 4 and 5). For *Wolbachia*, we first compared 15 OTU on a sequence length of 370 bp corresponding to *Wolbachia* sequences from clades A-F found in various hosts (S1 Table). It showed two groups of *Wolbachia* in our study. Thirteen OTU retrieved in *A*. *bilineata* and *D*. *radicum* samples (and retrieved with few sequences in *A*. *bipustulata* samples and *T*. *rapae* samples) belong to clade A and are related to the *Wolbachia* strain found in *Drosophila sp*. Two OTU retrieved in *A*. *bipustulata* from each population fall within Wolbachia clade B (Fig 3).

**Fig 6:**
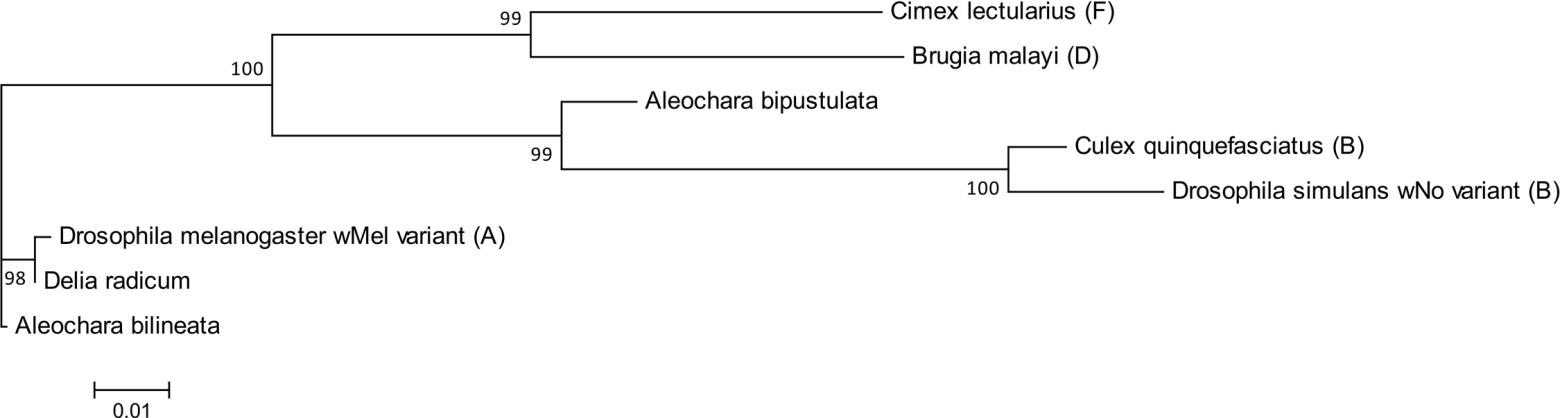
Maximum likelihood tree of Wolbachia spp. obtained from 16S V4-V5, 16S wspec and fbpA fragments. The Wolbachia hosts and the supergroup of the Wolbachia (when available) are indicated. Bootstrap values are given for each branch. Scaling is expressed in the proportion of substituted bases per site.

To increase resolution regarding the Wolbachia strains found in this study, we amplified another 415bp region of the 16S rDNA gene of Wolbachia using primers W-Spec, as well as a 478bp fragment of the fbpA gene using fbpA primers in two independent PCR (one per gene) using the DNA pool of each population. No amplification was found in *T*. *rapae* but one sequence per species was consistently recovered in *D*. *radicum*, *A*. *bilineata* and *A*. *bipustulata*. Our results confirm that the variant found in *A*. *bilineata* is very close from that of its host *D*. *radicum* (100% identity in the W-Spec sequence) but that it is not identical (5 differences in the fbpA sequence), which excludes a recent horizontal transfer. We also confirm that the *Wolbachia* variants of *A*. *bilineata* and *A*. *bipustulata* are unrelated (respectively 6 and 44 differences in the W-Spec and fbpA sequences). A phylogenetic tree built using 16S v4-v5, 16S W-Spec and fbpA sequences (Fig 6) shows that both the *Wolbachia* variant of *D*. *radicum* and that of *A*. *bilineata* belong to supergroup A. It also shows that the *Wolbachia* strain found in *A*. *bipustulata* is distinct from supergroups A, D and I and appears related to supergroup B.

Five OTUs identified as *Rickettsia*, respectively one in *A*. *bilineata* (central) samples and four in (western) *T*. *rapae*, were compared to *Rickettsia* sequences found in GenBank, the comparison being based on 375 bp. (S1 Table). The different groups of *Rickettsia* are not well supported by bootstrap values but strains infecting each insect host seem to be different: *A*. *bilineata* is infected by a *Rickettsia* strain in the Bellii group while *T*. *rapae* sequences are more closely related to *Rickettsia limonae* and to endosymbionts of the parasitoid wasp *Asobara tabida*. (Fig 4). Eleven OTU retrieved in the two *Aleochara* samples (and a few sequences retrieved in *T*. *rapae* and *D*. *radicum* samples) belonging to *Spiroplasma* were compared to several sequences found on GenBank using a 365 bp length (S1 Table). Ten OTU retrieved in the four species are closely related to *Spiroplasma* of the weevil *Curculio elephas* while one OTU found in *A*. *bilineata* from the central population was more closely related to the *Spiroplasma* strain infecting *Aedes* sp. mosquitoes. The partial 16S sequences are however too short and not variable enough to finely differentiate strains infecting each species and assess the possibility of horizontal transfers (Fig 5).

## Discussion

In the present study, we describe the bacterial communities associated with the adults of four interacting insect species, providing to our knowledge the first description of coleopteran parasitoids bacterial communities (*Aleochara sp*.) and of the microbiote of the parasitoid wasp *T*. *rapae*. We also bring the culture-independent data about the (so far undescribed) microbiota of *D*. *radicum*, a phytophagous insect which is also an important crop pest. A key aspect of our study is that we compared the bacterial communities of species related by host/parasitoid relationships and found that several OTUs (including different vertically transmitted symbiont species) are shared between at least two insect hosts, suggesting the possibility of horizontal transfers through trophic relationships. However, the high proportion of bacterial genera that are *not* shared between *D*. *radicum* and its three natural enemies show that the bacteriome of the host is clearly not the main source shaping the bacterial communities of its parasitoids. Moreover, possible horizontal transfers suggested by some of our results would require confirmation because 16S rDNA gene sequence data do not provide a good enough phylogenetic resolution to finely characterize bacterial strains, as shown by the exclusion of a putative horizontal transfer of *Wolbachia* between *D*. *radicum* and *A*. *bilineata* when examining the more variable fbpA gene.

### Bacterial diversity

Bacterial phyla identified in our study were the same as those identified in other animal models such as mammals [75], [76], reptiles [77], birds [78] or insects [13], [16], [79], [80] but because microbial DNA was isolated from whole bodies of insects, we cannot draw conclusions about the tissue specificity of the microbes observed in our species. Using a 97% homology criterion, we identified 1506 putative bacterial species from five phyla giving 108 different genera, which is comparable to bacterial diversity values reported in other insect models [13], [15], [48], [81]. *Trybliographa rapae* shows a highly diversified community compared to other parasitoid wasps [12] even though our laboratory adult *T*. *rapae* were fed honey, which has some antibiotic properties [82]. This diversity is probably due in part to our large sampling with DNA extracts from 20–24 individuals pooled in each sample, and it remains moderate when compared to that of termites [37]. On the other hand, the Chao1 estimator shows that despite our deep sampling (3000 sequences per sample) and the large number of identified OTUs, we probably covered less than half of insect bacterial communities for every sample except for the eastern population of *D*. *radicum* which has a diversity comparable to other studies on insects, with around 16 identified genera [12]. Some rare bacteria present at a low density in our samples may have been missed due to the high density of bacteria dominating each sample, which would explain that equitability is relatively low. A low equitability can result from a low number of reads [83]. However, this can be ruled out in our study: we obtained about 3000 sequences per sample which presumably allows to give a good idea of the natural abundance of bacteria in our samples. The number of reads can be biased if primers show significantly more affinity to some bacterial phylotypes [84]. Some contaminations can be observed in microbiota studies of phytophagous insects, where some reads correspond to chloroplastic DNA from plants [80]. In this study, however, no OTU was common to all samples and only a few were shared between the four species at least in one population. In addition, the bacterial communities we found are much more similar within species than between species and the microbiota from laboratory-reared parasitoids are not closer to that of their common rearing host (*D*. *radicum* from the eastern population) than to that of western *D*. *radicum* (which was not used in laboratory rearing). Accordingly, we think that trophic contaminations were limited in our samples. The normalization of results led to remove about a half of the sequence reads obtained during sequencing, but such homogenization of library sizes has been shown to be important to compare bacterial communities of different environments [85].

Three bacterial genera are widespread among our samples and are also known to be maternally transmitted bacteria. *Wolbachia sp*. dominated *D*. *radicum* bacterial communities and was also detected in all but one sample (eastern *T*. *rapae*) in the three other species. This *Alphaproteobacterium* is the most frequent symbiont in insects and it also infects a large number of arthropod species, often manipulating the reproduction of its host [44]. Low detection in *T*. *rapae* (only three sequences) was not confirmed when we used fbpA or W-Spec primers, so the presence of *Wolbachia* in this species remains dubious at this stage. *Rickettsia sp*. dominated the western *T*. *rapae* sample and was also detected at low frequencies in the two wild *A*. *bilineata* populations sampled. *Rickettsia sp* is a frequent insect endosymbiont. First known as pathogens of mammals vectored by insects, these bacteria are today recognized to induce (like *Wolbachia*) reproductive alterations and fitness effects in their hosts [86], [87], [88]. The *Tenericutes Spiroplasma sp*. is also known as a facultative endosymbiont in insects; these bacteria are detected in every *Aleochara* sample and seem to particularly dominate *A*. *bipustulata* communities; they are also detected in *D*. *radicum* (four sequences) and in western *T*. *rapae* (three sequences). *Spiroplasma sp*. are associated both endocellularly and extracellularly with a variety of plants and arthropods. They manipulate reproduction of their hosts such as male-killing phenotypes [89] but positive fitness effects (protection against parasitic wasps and fungal diseases) have also been described [90], [91]. The *Gammaproteobacteria* of the genus *Stenotrophomonas sp*. and the *Firmicutes* of the genus *Bacillus sp*. are also strongly represented, mostly in the two wild *A*. *bilineata* samples but also in *T*. *rapae* and *A*. *bipustulata* laboratory samples. A bacterium of the genus *Stenotrophomonas* has been recently described to protect its host, the fly *Stomoxys calcitrans*, against *Beauveria bassiana* an entomopathogenic fungus [92]. These bacteria are described as environmental bacteria present in water and soils but they are also described as opportunistic gut bacteria that can invade insect guts and prevent the invasion by other pathogenic bacteria [8]. Other genera are less represented in terms of number of sequences but are shared by at least two species in our study. *Vagococcus sp*., *Staphylococcus sp*., *Sporosarcina sp*., *Ochrobactrum sp*., *Carnobacterium sp*., *Burkholderia sp*., *Ralstonia sp*., *Delftia sp*., *Achromobacter sp*. are all commonly present environmentally and in insect guts [45]. Another *Alphaproteobacteria*, *Asaia sp*. is strongly represented in *T*. *rapae*, dominating the eastern population sample. *Asaia sp*. is an acetic acid bacterium, which has been described to invade digestive tracts of mosquitoes (where they also invade reproductive tracts; [93], plant hoppers [94] and ants [95]. The roles and the fitness effects of *Asaia sp*. on its hosts remain uncertain; other acetic acid bacteria such as *Gluconacetobacter sp*. (detected in eastern *D*. *radicum*) could also be potential endosymbionts [96].

### Comparison of bacterial communities

Although samples harboured very different microbial communities in terms of composition, respectively 21% of bacterial genera identified in *A*. *bilineata* (8/37), 25% for *D*. *radicum* (5/20), 33% for *A*. *bipustulata* (11/33) and 38% for *T*. *rapae* (29/76) were shared by the two populations sampled, giving an idea of the core microbiota of each species. In the same way, only a few OTU were shared between at least two species and the comparison of bacterial communities based on their UniFrac distance clustered as follows: (i) the two replicates of each sample, demonstrating the reproducibility of the sequencing method used, then (ii) samples from the same species and finally, (iii) *Aleochara* sp. samples, showing that the main environmental filter influencing the microbial communities in our samples is probably phylogenetic proximity. Host phylogeny has already been shown to explain much of the variation found in insect bacterial communities [16]. Another important factor driving the bacterial community of many insects is diet [15], [97], but it was not apparent in our study. Indeed, judging by their bacterial communities, *Aleochara* sp. were more distant from another parasitoid (*T*. *rapae*) sharing their diet during its development than from their common phytophagous host *D*. *radicum*. However, *Aleochara* sp. and *T*. *rapae* have very different adult diets (adult staphylinids are opportunistic predators and scavengers while adults of *T*. *rapae* feed on flowers) and bacterial communities can change markedly during larval development [12]. It could explain the differences observed here between coleopteran and hymenopteran parasitoids. Finally, even if geographical location seems to induce some variation in the microbial communities of each species, it was not a major driver of the bacterial community composition at the scale used in our study (a span of 150 km).

The proximity between communities found in the two *Aleochara* species is interesting because *A*. *bilineata* samples were directly sampled in fields and were killed as soon as they emerged from their host while *A*. *bipustulata* individuals were adults sampled from a rearing cage and came from a stock reared in the laboratory for more than ten generations using eastern *D*. *radicum* as a host. Two opposite hypotheses could explain such a similarity between two bacterial communities: (i) the two *Aleochara* species share a similar core microbiota in the wild and rearing them in the laboratory does not modify it significantly, or (ii) the generalist *A*. *bipustulata* has a microbiota markedly different from that of the specialist *A*. *bilineata* in the wild, but this community was modified by rearing it on *D*. *radicum* pupae only, leading to a "specialist diet". The lack of freshly wild-captured *A*. *bipustulata* samples in our study does not allow us to settle this case, but the potential of laboratory rearing to modify microbiota is known. For example Lehman et al. [10] found that laboratory rearing reduced the bacterial diversity in the beetle *Poecillus chalcites*. Temperatures in the laboratory are also more regular and most of the year higher than those experienced in the fields sampled, which can raise the densities of several facultative or obligate endosymbionts like *Wolbachia* [98] or *Buchnera* [99]. Accordingly, the favourable rearing conditions might explain at least in part the dominance of sequences corresponding to endosymbionts in our laboratory samples.

### Phylogenetic analysis of heritable endosymbionts

Three known facultative and maternally transmitted endosymbionts are shared by at least two species in our study and they show very different patterns of phylogenetic proximity. In *Wolbachia*, the combining of 16SrDNA (W-Spec) and fbpA sequences allows to describe with confidence at least three *Wolbachia* variants, two from supergroup A and another from a distinct supergroup, possibly B. One variant from supergroup A is found in *D*. *radicum* while the other A-group *Wolbachia* is found in its specialist parasitoid *A*. *bilineata*. The two variants are very closely related but not identical. Accordingly, horizontal transfer might have occurred between host and parasitoid, but if so it was not a recent one. On the other hand, the variant found in *A*. *bipustulata* appears related to supergroup B (Fig 3). Therefore, it is vastly distinct both from that of its host *D*. *radicum* and from that of the closely related parasitoid *A*. *bilineata*. None of these results is surprising since the phylogeny of *Wolbachia* is known to be incongruent with that of its arthropod hosts [24], [44], precisely because horizontal transfers between species are not uncommon at a large phylogenetic scale. For *Rickettsia*, two phylogenetically distinct groups of sequences have been identified by 454 sequencing, one in *A*. *bilineata* samples and one in *T*. *rapae* samples. Finally, two groups of *Spiroplasma sp*. sequences have been identified, one exclusive to eastern *A*. *bilineata* samples while the second is shared between all samples except eastern *T*. *rapae*. Such shared OTU raise again the possibility of past horizontal transfers between species but this hypothesis cannot be confirmed at this stage because the 16S rDNA gene we used shows a limited variability, and infection by several strains of vertically transmitted symbionts has been described in several insect models [73, 74].

## Conclusions and Perspectives

The description of the rich bacterial communities linked to the host/parasitoid associations we found here is a first step toward further experimentation. Some bacterial genera known to impact fitness of insects have been identified. Especially, the presence of three distinct vertically transmitted endosymbionts in such a small trophic network was unexpected. The impact of these endosymbionts on the fitness of their hosts should be investigated. Meanwhile, our results suggest that trophic relationships between hosts and their parasitoids, however intimate, might not weight much in the face of phylogenetic proximity when it comes to shaping insect microbiota.

## Supporting Information

S1 Fig **Rarefaction curves showing the number of detected MOTUs as a function of the number of high quality reads in *Delia radicum* (A), *Trybliographa rapae* (B), *Aleochara bilineata* (C) and *Aleochara bipustulata* (D).** Coding of populations sampled and homology thresholds (95%, 97% and 99%): see legend included in the figure.
(TIF)

S1 Table Reference sequences of 16S rDNA genes of *Wolbachia*, *Spiroplasma*, *Rickettsia*, *Ehrlichia* and *Mycoplasma*. (DOCX)
