# Supplementary material for: Bacterial Community Diversity Harboured by Interacting Species
Source: PLoS One. 2016 Jun 3;11(6):e0155392. doi: 10.1371/journal.pone.0155392 (PMC4892616; doi:10.1371/journal.pone.0155392)
Supplement: S1 Table — (DOCX) [file pone.0155392.s002.docx]

**Supplementary data**

**S1 Table. Reference sequences of 16S rDNA genes corresponding to *Wolbachia*, *Spiroplasma, Rickettsia*, *Ehrlichia* and *Mycoplasma***.

| Accession number | Bacteria | Host species | Clade |
| --- | --- | --- | --- |
| gi\|149136\|gb\|M84687.1\| | *Wolbachia sp.* | *Nasonia vitripennis* | A |
| gi\|862345\|gb\|L02882.1\| | *Wolbachia sp.* | *Muscidifurax uniraptor* | A |
| gi\|895916\|emb\|X87407.1\| | *Wolbachia sp.* | *Aphytis diaspidis* | A |
| gi\|89146850\|gb\|DQ399339.1\| | *Wolbachia sp.* | *Afrocimex constrictus* | A |
| gi\|29469648\|gb\|AY227742.1\| | *Wolbachia sp.* | *Drosophila simulans strain wAu* | A |
| gi\|89515290\|gb\|DQ412085.1\| | *Wolbachia sp.* | *Drosophila simulans strain wRi* | A |
| gi\|89515288\|gb\|DQ412083.1\| | *Wolbachia sp.* | *Drosophila melanogaster strain wMel* | A |
| gi\|2565104\|gb\|U83092.1\| | *Wolbachia sp.* | *Gryllus rubens* | B |
| gi\|862346\|gb\|L02883.1\| | *Wolbachia sp.* | *Trichogramma cordubensis spain isolate* | B |
| gi\|48500\|emb\|X61768.1 | *Wolbachia sp.* | *Culex pipiens* | B |
| gi\|510558\|emb\|X65674.1\| | *Wolbachia sp.* | *Tribolium confusum* | B |
| gi\|149138\|gb\|M84689.1\| | *Wolbachia sp.* | *Nasonia giraulti* | B |
| gi\|666437130\|gb\|KJ814199.1\| | *Wolbachia sp.* | *Armadillidium vulgare clone A* | B |
| gi\|2565102\|gb\|U83090.1\| | *Wolbachia sp.* | *Gryllus pennsylvanicus* | B |
| gi\|9857235\|emb\|AJ276499.1\| | *Wolbachia sp.* | *Onchocerca gibsoni* | C |
| gi\|9857234\|emb\|AJ276498.1\| | *Wolbachia sp.* | *Onchocerca gutturosa* | C |
| gi\|29134865\|gb\|AF487892.1\| | *Wolbachia sp.* | *Dirofilaria immitis strain DAX* | C |
| gi\|9857236\|emb\|AJ276500.1 | *Wolbachia sp.* | *Dirofilaria repens* | C |
| gi\|2952471\|gb\|AF051145.1\| | *Wolbachia sp.* | *Brugia malayi* | D |
| gi\|44804337\|emb\|AJ548798.1\| | *Wolbachia sp.* | *Litomosoides hamletti* | D |
| gi\|6469659\|gb\|AF179630.1\| | *Wolbachia sp.* | *Folsomia candida* | E |
| gi\|21322700\|emb\|AJ422184.1\| | *Wolbachia sp.* | *Mesaphorura macrochaeta* | E |
| gi\|18076426\|emb\|AJ292347.1\| | *Wolbachia sp.* | *Microcerotermes sp.* | F |
| gi\|175827\|gb\|M85267.1\| | *Wolbachia sp.* | *Rhinocyllus conicus* | F |
| gi\|34978559\|gb\|AY316361.1\| | *Wolbachia sp.* | *Cimex lectularius* | F |
| gi\|54633739\|gb\|AY764279.1 | *Wolbachia sp.* | *Zootermopsis angusticollis* | H |
| gi\|60098027\|emb\|AJ628416.1\| | *Wolbachia sp.* | *Ctenocephalides felis* | I |
| gi\|44804341\|emb\|AJ548802.1\| | *Wolbachia sp.* | *Dipetalonema gracile* | J |
| gi\|369727448\|gb\|JN384095.1\| | *Wolbachia sp.* | *Toxoptera aurantii strain B* | K |
| gi\|212550177\|gb\|EU833482.1\| | *Wolbachia sp.* | *Radopholus similis* | L |
| gi\|369727373\|gb\|JN384057.1\| | *Wolbachia sp.* | *Cavariella sp* | M |
| gi\|52355655\|gb\|AY620430.1\| | *Wolbachia sp.* | *Cinara cedri* | N |
| gi\|6435827\|gb\|AF147752.2\| | *Ehrlichia chaffeensis* |  |  |
|  |  |  |  |

| référence | species name | host | serological group |
| --- | --- | --- | --- |
| gi\|110815963\|gb\|AF443616.3\| | *Mycoplasma hominis* | Human |  |
| gi\|296005972\|gb\|HM015669.1\| | *Spiroplasma citri* | *citrus* sp. | I-1 |
| gi\|219846166\|ref\|NR_025756.1\| | *Spiroplasma melliferum* | *Honey bees* | I-2 |
| gi\|37782190\|gb\|AY189133.1\| | *Spiroplasma insolitum* | plant | I-6 |
| gi\|343206085\|ref\|NR_044672.1\| | *Spiroplasma poulsonii* | *Drosophila willistoni* | II |
| gi\|37782188\|gb\|AY189131.1\| | *Spiroplasma floricola* | *Liriodendron tulipifera* | III |
| gi\|559795267\|ref\|NR_104858.1\| | *Spiroplasma apis* | Bees, flowers | IV |
| gi\|559795362\|ref\|NR_104955.1\| | *Spiroplasma mirum* | Rabbit ticks | V |
| gi\|37782196\|gb\|AY189309.1\| | *Spiroplasma syrphidicola* | *Eristalis arbustorum* | VIII-1 |
| gi\|37782184\|gb\|AY189127.1\| | *Spiroplasma chrysopicola* | *Chrysops* sp. | VIII-2 |
| gi\|37782186\|gb\|AY189129.1\| | *Spiroplasma culicicola* | *Aedes mosquito* | X |
| gi\|37782198\|gb\|AY189311.1\| | *Spiroplasma velocicrescens* | *Monobia quadridens* | XI |
| gi\|559795164\|ref\|NR_104751.1\| | *Spiroplasma diabroticae* | *Diabrotica undecimpunctutata* | XII |
| gi\|37782195\|gb\|AY189308.1\| | *Spiroplasma sabaudiense* | *Aedes mosquito* | XIII |
| gi\|37782185\|gb\|AY189128.1\| | *Spiroplasma corruscae* | *Ellychnia corrusca* | XIV |
| gi\|112785087\|gb\|DQ861914.1\| | *Spiroplasma cantharicola* | Cantharid beetle | XVI-1 |
| gi\|37782197\|gb\|AY189310.1\| | *Spiroplasma turonicum* | *Haematopota sp.* | XVII |
| gi\|37782193\|gb\|AY189306.1\| | *Spiroplasma litorale* | *Tabanus nigrovittatus* | XVIII |
| gi\|37782191\|gb\|AY189134.1\| | *Spiroplasma lampyridicola* | *Photuris pennsylvanicus* | XIX |
| gi\|37782192\|gb\|AY189305.1\| | *Spiroplasma leptinotarsae* | *Colorado beetle* | XX |
| gi\|37782183\|gb\|AY189126.1\| | *Spiroplasma chinense* | *Calystegia hederacea* | XXIV |
| gi\|37782187\|gb\|AY189130.1\| | *Spiroplasma diminutum* | *Culex mosquito* | XXV |
| gi\|37782182\|gb\|AY189125.1\| | *Spiroplasma alleghenense* | *Panorpa helena* | XXVI |
| gi\|112418367\|gb\|DQ860100.1\| | *Spiroplasma lineolae* | *Tabanus lineola* | XXVII |
| gi\|37782194\|gb\|AY189307.1\| | *Spiroplasma montanense* | *Hybomitra opaca* | XXXI |
| gi\|37782189\|gb\|AY189132.1\| | *Spiroplasma helicoides* | tabanid flies | XXXII |
| gi\|559795166\|ref\|NR_104753.1\| | *Spiroplasma tabanidicola* | tabanid flies | XXXIII |
| gi\|323367212\|gb\|JF266577.1\| | *Spiroplasma sp.* | *Agathemera claraziana* | not related |
| gi\|124481969\|gb\|EF121346.1\| | *Spiroplasma sp.* | *Ctenocephalides felis* | not related |
| gi\|292668056\|gb\|GU815127.1\| | *Spiroplasma sp.* | *Harpalus pennsylvanicus* | not related |
| gi\|50830969\|gb\|AY569829.1\| | *Spiroplasma sp.* | *Fannia manicata* | not related |
| gi\|6978971\|dbj\|AB030022.1\| | *Spiroplasma sp.* | *Antonina crawii* | not related |
| gi\|387865305\|gb\|JQ692307.1\| | *Spiroplasma sp.* | *Curculio glandium* | not related |
| gi\|294345274\|dbj\|AB553862.1\| | *Spiroplasma sp.* | *Laodelphax striatellus* | not related |
| gi\|387865304\|gb\|JN100091.1\| | *Spiroplasma* sp. | *Curculio elephas* | not related |
| gi\|4582256\|emb\|AJ132412.1\| | *Spiroplasma* sp. | *Harmonia axyridis* | not related |
| gi\|410992257\|gb\|JX943566.1\| | *Spiroplasma* sp. | *Acyrthosiphon pisum* | not related |

| Références | Species name | Host/Vector | Group of Rickettsia |
| --- | --- | --- | --- |
| gi\|538440\|gb\|L36221.1\| | *Rickettsia typhi* | *Xenopsylla cheopis* | typhus fever |
| gi\|152479\|gb\|M21789.1\| | *Rickettsia prowazekii* | Ticks | typhus fever |
| gi\|68525480\|gb\|DQ062433.1\| | *Rickettsia peacockii strain Rustic* | Ticks | spotted fever |
| gi\|538432\|gb\|L36213.1\| | *Rickettsia japonica strain YM* | Ticks | spotted fever |
| gi\|310974984\|ref\|NR_036848.1\| | *Rickettsia sibirica strain 246* | Ticks | spotted fever |
| gi\|556410\|gb\|L36673.1\| | *Rickettsia parkeri* | Ticks | spotted fever |
| gi\|535752\|gb\|L36105.1\| | *Rickettsia conorii* | *Rhipicephalus sanguineus* | spotted fever |
| gi\|535745\|gb\|L36098.1\| | *Rickettsia africae* | *Amblyomma variegatum* | spotted fever |
| gi\|4501837\|gb\|AF060705.2\| | *Rickettsia honei strain RB* | Ticks | spotted fever |
| gi\|1311468\|dbj\|D84558.1\| | *Rickettsia sp.* | *Ixodes scapularis* | spotted fever |
| gi\|225547930\|gb\|FJ609406.1\| | *Rickettsia sp.* | *Aulogymnus balani* strain ABSWASP | transitional |
| gi\|600168\|gb\|U17644.1\| | *Rickettsia australis* | Ticks | transitional |
| gi\|109727229\|gb\|DQ652592.1\| | *Rickettsia sp.* | *Liposcelis bostrychophila* strain London | transitional |
| gi\|73671367\|gb\|DQ102712.1\| | *Rickettsia felis strain scc50* | *Ctenocephalides felis* | transitional |
| gi\|535746\|gb\|L36099.1\| | *Rickettsia akari* | Mites | transitional |
| gi\|86475999\|dbj\|AB231472.1\| | *Rickettsia sp.* | *Neochrysocharis formosa* | transitional |
| gi\|225547931\|gb\|FJ609407.1\| | *Rickettsia sp.* | *Pediobius rotundatus* strain PRWASP | transitional |
| gi\|555998\|gb\|U15162.1\| | *Rickettsia canadensis* | Ticks | canadensis |
| gi\|66774597\|gb\|AY961085.1\| | *Rickettsia sp.* | *Coccotrypes dactyliperda* | adalia |
| gi\|450812\|gb\|U04163.1\| | *Rickettsia sp.* | *Adalia bipunctata* | adalia |
| gi\|225547922\|gb\|FJ609398.1\| | *Rickettsia sp.* | *Subcoccinella vigintiquatuorpunctata* strain J | adalia |
| gi\|225547926\|gb\|FJ609402.1\| | *Rickettsia sp.* | *Halyzia sedecimguttata* strain D | adalia |
| gi\|33340523\|gb\|AF322443.1\| | *Rickettsia limoniae strain Brugge* | *Macrolophus caliginosus* | Torix |
| gi\|38175202\|dbj\|AB113214.1\| | *Rickettsia sp.* | *Torix tukubana* | Torix |
| gi\|38175203\|dbj\|AB113215.1\| | *Rickettsia sp.* | *Hemiclepsis marginata* | Torix |
| gi\|58978283\|gb\|AY753175.1\| | *Rickettsia sp.* | *Tetranychus urticae* clone pAJ252 | bellii |
| gi\|1147763\|gb\|U42084.1\| | *Rickettsia sp.* | *Acyrthosiphon pisum* strain PAR | bellii |
| gi\|71082704\|gb\|DQ077707.1\| | *Rickettsia sp.* | *Bemisia tabaci* | bellii |
| gi\|535750\|gb\|L36103.1\| | *Rickettsia bellii* | *Amblyomma sp.* | bellii |
| gi\|365811827\|gb\|JN182552.1\| | *Rickettsia sp.* | *Pnigalio soemius* isolate PS_CS2 | bellii |
| gi\|559795286\|ref\|NR_104877.1\| | *Rickettsia hoogstraalii* | Ticks | not found |
| gi\|506787\|gb\|U11021.1\| | *Rickettsia rickettsii* | Ticks | not found |
| gi\|224495120\|gb\|FJ603467.1\| | *Rickettsia sp.* | *Asobara tabida* clone At-R 16S | not found |
| gi\|538433\|gb\|L36214.1\| | *Rickettsia massiliae strain Mtu1* | *Rhipicephalus turanicus* | not found |
| gi\|193782788\|gb\|EU272189.1\| | *Candidatus Rickettsia barbariae* | Ticks | not found |
| gi\|6435827\|gb\|AF147752.2\| | *Ehrlichia chaffeensis* |  |  |
